# Simon’s Clinical Diagnosis

**Published:** 1900-11

**Authors:** 


Simon's Clinical Diagnosis. A Manual of Clinical Diagnosis by Microscopical and Clinical Methods. (For Students, Hospital Physicians and Practitioners. By Charles E. Simon, M. D., Late Assistant Resident Physician Johns-Hopkins Hospital, Baltimore. In one very handsome octavo volume of 563 pages, with 136 engravings and 18 full-page colored plates. Cloth, $3.50 net. Lea Brothers & Co., Philadelphia and New York.

Three new editions of this book have been called for in four years, which shows how much the best methods of clinical diagnosis are appreciated. No practitioner can afford to pass by the modern methods of diagnosis. The laboratory and the microscope must claim a large share of his attention. More exact knowledge is required to give greater precision to his work. If he cannot afford the time to study such books of Simon's he must drop out of the procession.

